# Preparation and Evaluation of Coal Fly Ash/Chitosan Composites as Magnetic Supports for Highly Efficient Cellulase Immobilization and Cellulose Bioconversion

**DOI:** 10.3390/polym10050523

**Published:** 2018-05-14

**Authors:** Limin Zang, Xuan Qiao, Lei Hu, Chao Yang, Qifan Liu, Chun Wei, Jianhui Qiu, Haodao Mo, Ge Song, Jun Yang, Chanjuan Liu

**Affiliations:** 1 College of Material Science and Engineering, Guilin University of Technology, Guilin 541004, China; 2016034@glut.edu.cn (L.Z.); qiaoxuan150103@gmail.com (X.Q.); hlpolymer010@gmail.com (L.H); liuqifan0108@gmail.com (Q.L.); 1986024@glut.edu.cn (C.W.); SongGe0730@gmail.com (G.S.); yjun013@gmail.com (J.Y.); 2 Department of Machine Intelligence and Systems Engineering, Faculty of Systems Science and Technology, Akita Prefectural University, Yurihonjo 015-0055, Japan; qiu@akita-pu.ac.jp (J.Q.); D18S003@akita-pu.ac.jp (H.M.)

**Keywords:** magnetic composites, chitosan, coal fly ash, enzyme immobilization

## Abstract

Two magnetic supports with different morphologies and particle sizes were designed and prepared for cellulase immobilization based on chitosan and industrial by-product magnetic coal fly ash (MCFA). One was prepared by coating chitosan onto spherical MCFA particles to form non-porous MCFA@chitosan gel microcomposites (Support I) with a size of several micrometers, and the other was prepared using the suspension method to form porous MCFA/chitosan gel beads (Support II) with a size of several hundred micrometers. Cellulase was covalent binding to the support by glutaraldehyde activation method. The morphology, structure and magnetic property of immobilized cellulase were characterized by scanning electron microscopy, Fourier transform infrared spectroscopy and a vibrating-sample magnetometer. The cellulase loading on Support I was 85.8 mg/g with a relatlvely high activity recovery of 76.6%, but the immobilized cellulase exhibited low thermal stability. The cellulase loading on Support II was 76.8 mg/g with a relative low activity recovery of 51.9%, but the immobilized cellulase showed high thermal stability. Cellulase immobilized on Support I had a glucose productivity of 219.8 mg glucose/g CMC and remained 69.9% of the original after 10 cycles; whereas the glucose productivity was 246.4 mg glucose/g CMC and kept 75.5% of its initial value after 10 repeated uses for Support II immobilized cellulase. The results indicate that the two supports can be used as cheap and effective supports to immobilize enzymes.

## 1. Introduction

Because of the high efficiency and specificity in catalyzing reactions under mild conditions, enzymes have gained widespread attention and have been widely exploited in the fields of biotechnology, medicine, environmental protection and food industries [1,2,3,4]. However, there are drawbacks that cannot be ignored, including difficulty in recovery and reuse, poor stability, and relatively high cost which restrict their application. The previous studies have proved that immobilization of enzymes by some strategies such as adsorption, covalent bonding, entrapment, and encapsulation could reuse enzymes, improve stability, reduce inhibition problems, improve selectivity or specificity, and in certain cases obtain higher activity [5,6,7,8,9,10,11].

As an industrial by-product, coal fly ash (CFA) is generated in large amounts with the development of coal fire plants, which poses serious hazards to human health and the environment [12,13]. Considerable research has been focused on its utilization, such as construction materials [14], soil amendment [15] and sorbents [16]. During combustion, iron in coals is transformed into magnetite, hematite, maghemite, Fe^2+^-silicate, Fe^3+^-silicate, etc., forming the magnetic fractions [17,18]. The magnetic fractions derived from CFA capture our attention and are considered to be an ideal candidate as a support for enzyme immobilization material because of the convenience in recovery which can be easily separated from the reaction system by magnets. However, directly using original magnetic coal fly ash (MCFA) as a support would result in low loading amount and suffer from enzyme leaching due to the weak interactions between the enzyme and MCFA. Thus, to attempt to improve linkages between the enzyme and MCFA while maintain bioactivity of the enzyme is particularly important. Surface modification of magnetic supports using biocompatible material with abundant functional groups has proved to be a practicable approach [19,20].

Chitosan is a natural polyaminosaccharide obtained from chitin by deacetylation, which possesses many advantages, including good biocompatibility, remarkable affinity to proteins, nontoxicity, low cost and the presence of functional groups (amino, hydroxyl and hydroxymethyl groups) [6,21,22,23]. Given the significant advantages, chitosan is considered as an ideal support for enzyme immobilization, and various chitosan-based supports in different forms have been developed, such as beads, membranes, microspheres, fibers or sponges [24,25,26]. Chitosan is soluble in acidic solutions due to the primary amino groups in chitosan with a pKa value of ~6.5, which means chitosan is protonated below pH 6.5 [25,27]. The dependence of soluble–insoluble transition on pH was exploited to modify negatively-charged metal oxide (Fe_3_O_4_, Al_2_O_3_, TiO_2_, etc.) or clays (attapulgite, halloysite, bentonite, montmorillonite, etc.) with via ionic exchanges [28,29,30,31,32,33,34].

Not only the nature but also the morphology of the support can affect the properties of immobilized enzymes. For non-porous supports, diffusion limitations do not occur since the enzyme is immobilized on the surface of the supports, which can be applied to large or solid substrates [35,36,37]. However, inactivation problems caused by interactions with hydrophobic interfaces should be taken into account [5,38]. The small particle size of non-porous supports is conducive to enhancing the enzyme loading capacity due to the enhanced specific surface area, but recovery of them may become difficult [5,35]. For porous supports, enzyme inactivation caused by interactions with hydrophobic interfaces can be prevented because most enzymes are immobilized inside the pores [5,35,38,39]. However, the use of porous supports is limited when the substrate is large or insoluble due to the diffusion problems [5]. In addition, the size of the pore should be proper, larger than the target protein at least. The larger pores may prevent the pores from being blocked by large contaminants, but decrease enzyme loading capacity.

MCFA/chitosan gel composites with two different morphologies and particle sizes were prepared via two strategies. One was non-porous MCFA@chitosan gel microcomposites with a size of several micrometers, and the other was porous MCFA/chitosan gel beads with a size of several hundred micrometers. Then, the as-prepared supports were employed to immobilize enzyme by using glutaraldehyde (GDA) activation method. The GDA activation of supports is a very versatile and popular technique for enzyme immobilization, although the activation mechanism is still under discussion [40]. GDA activation method has proved to be facile and efficient, and in certain cases it can improve enzyme stability by multipoint or multisubunit immobilization [41,42,43,44,45,46]. Cellulase which can effectively hydrolyze cellulose to produce glucose, was immobilized on these two supports to evaluate the performance of MCFA/chitosan gel composites as magnetic supports for enzyme immobilization. The influences of morphology and particle size on enzyme loading capacity, activity recovery, optimum pH and temperature, thermal stability and reusability of immobilized enzymes were studied.

## 2. Materials and Methods

### 2.1. Materials

Cellulase was bought from Meiji Seika Pharma Co., Ltd. (Tokyo, Japan). Magnetic coal fly ash (MCFA) powder was separated from the coal fly ash powder which was obtained from Guodian Yongfu Power Generation Co., Ltd. (Guilin, China). Chitosan and carboxy methyl cellulose sodium salt (CMC) were bought from Nacalai Tesque, Inc. (Kyoto, Japan). Acetic acid, glutaraldehyde (GDA, 50%, *v*/*v*), sodium hydroxide (NaOH), kerosene, Span-80 and Tween-80 were purchased from Aladdin Reagent Co., Ltd. (Shanghai, China).

### 2.2. Preparation of MCFA-Chitosan Supports

Two supports based on chitosan and MCFA were prepared for the immobilization of cellulase in this work.

Support I: MCFA@chitosan gel microcomposites were prepared according to previous work with some modification [47]. Firstly, 1 g chitosan was dissolved in 100 mL of 1% acetic acid aqueous solution under constant stirring. Then 4 g MCFA powder was added and the mixture was kept stirring for another 1 h to disperse the MCFA particles and the chitosan uniformly. Next, 50 mL of 1 M NaOH aqueous solution was added to form MCFA@chitosan gel microcomposites. The resulting product was magnetically separated and washed with distilled water. The MCFA@chitosan gel microcomposites were labelled as Support I.

Support II: MCFA/chitosan gel beads were prepared using the same MCFA/chitosan feeding ratio as Method I according to the previous work with some modification [48]. In brief, 4 g MCFA powder was added to 25 mL of chitosan solution (1 g chitosan dissolved in 25 mL of 1% acetic acid aqueous solution) and the mixture was kept stirring for 1 h to get homogenization. Later, the mixture was added dropwise into the oil phase of kerosene (50 mL) along with 1% (*w*/*v*) Span-80 and 0.3% Tween-80 (*w*/*v*) as stabilizers while being stirred (600 rpm) at room temperature. The suspension was stirred with a mechanical stirrer at 600 rpm for a further 2 h to form spherical beads. Subsequently, the resulting beads were quickly transferred into a coagulation bath containing a mixture of 80 mL of NaOH solution (2 M) and 20 mL of ethanol. After immersion for 12 h, the MCFA/chitosan gel beads were magnetically separated and washed with distilled water repeatedly. The MCFA/chitosan gel beads were labelled as Support II.

### 2.3. Immobilization of Cellulase

The above-obtained supports were used to immobilize cellulase by covalent bonding via GDA activation method with some modifications [29]. The as-prepared Support I was totally mixed with 120 mL of 2.5% GDA aqueous solution and the reaction was carried out at room temperature for 2 h. After magnetic separation, the GDA activated support was completely washed with distilled water several times. Subsequently, 100 mL of cellulase solution (6 mg/mL, dissolved in 0.1 M acetate buffer) was added and reacted with the GDA activated support. The immobilization reaction was performed at room temperature for 2 h with gentle stirring. The obtained immobilized cellulase was washed with 0.1 M acetate buffer repeatedly and labelled as Method I. Support II was also used for cellulase immobilization according to the above method, and the corresponding immobilized cellulase was labelled as Method II. The obtained immobilized cellulase was stored at 4 °C for subsequent enzyme assays.

Cellulase loading quantities were determined from the filtrate following the Bradford protein assay method using a Metash UV-9000S UV-vis spectrophotometer [49]. The cellulase immobilization yield was calculated from the following equation:

$$\mathrm{Immobilization}\;\mathrm{yield}\;\left(\%\right)\;=\;\frac{C_{0}V_{0}\;-\;C_{f}V_{f}}{C_{0}V_{0}}\times 100\%$$

where *C*_0_ and *C_f_* is the protein concentration of the initial cellulase solution before immobilization and the filtrate after immobilization, respectively. *V*_0_ and *V_f_* is the volume of the initial cellulase solution before immobilization and the filtrate after immobilization, respectively.

### 2.4. Characterization

The morphologies of the samples were observed using a field emission scanning electron microscope (SEM, Hitachi S-4800, Tokyo, Japan). The structures of the samples were studied by Fourier transform infrared (FT-IR, Nicolet Impact 400, Nicolet Instrument Technologies, Inc., Madison, WI, USA) spectra with a resolution of 4 cm^−1^. A vibrating-sample (VSM, Lake Shore 7307, Lake Shore Cryotronics, Westerville, OH, USA) magnetometer was used to analyze the magnetic properties of the samples.

### 2.5. Activity Assay

The cellulase activity was deduced as the hydrolysis of CMC according to the method of IUPAC with some modifications [50]. The typical activity assay was as follows: 1% CMC solution was prepared by dissolving CMC in 0.1 M acetate buffer (pH 4) in advance and used as substrate. For the free cellulase, 0.5 mL of 1% CMC solution was reacted with 0.5 mL of cellulase solution (0.005 mg/mL, dissolved in 0.1 M acetate buffer, pH 4). For the immobilized cellulase, the known weight of immobilized cellulase was dispersed to 25 mL of 0.1 M acetate buffer (pH 4) and immersion for 5 min, and the concentration of cellulase was equal to 0.005 mg/mL. Then 25 mL of 1% CMC solution was added to proceed the hydrolysis. The amount of glucose produced in 30 min at 50 °C was used to evaluate the cellulase activity which was measured by a UV-vis spectrophotometer at 540 nm using DNS as the reagent. When investigating the effect of pH on activity, 1% CMC solution was prepared by dissolving CMC in proper pH buffer (0.1 M), and the free and immobilized cellulase solutions were prepared using the corresponding pH buffer. In this study, each experiment was performed in triplicate to obtain an average.

### 2.6. Determination of Thermal Stability

The thermal stability of free and immobilized cellulase was studied by incubating them in 0.1 M acetate buffer (pH 4) at 70 °C. The residual activities of free and immobilized cellulase were measured at different time intervals.

### 2.7. Reusability Assay

Taking a “practical application” perspective, the immobilized cellulase was used to hydrolyze 1% CMC for 24 h. After magnetic separation, the immobilized cellulase was reacted with a fresh 1% CMC solution for another hydrolysis process. The glucose produced during 24 h at each cycle was measured to evaluate the reusability.

## 3. Results and Discussion

### 3.1. Strategy for Preparation of Supports

**Figure 1:**
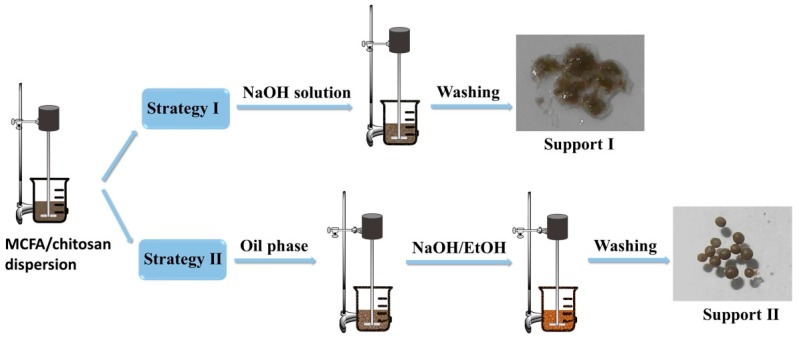
Schematic illustration of preparation process of MCFA/chitosan supports.

**Figure 2:**
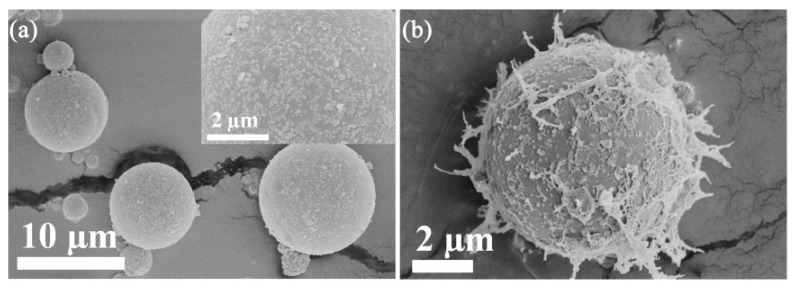

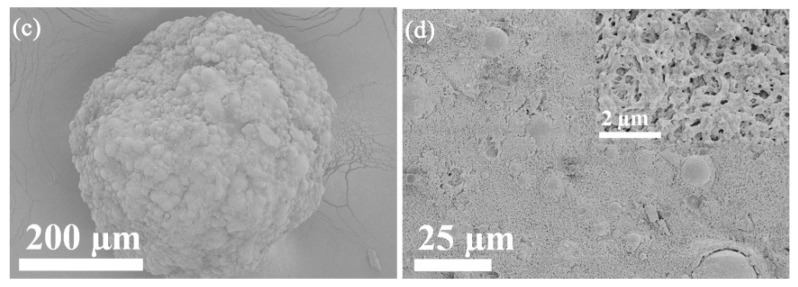
SEM micrographs of (**a**) pristine MCFA, (**b**) Support I and (**c**,**d**) Support II.

Two strategies were adopted to prepare MCFA/chitosan supports with entirely different morphologies and particle sizes. The preparation process is illustrated in Figure 1. In the first strategy, MCFA powder was dispersed in chitosan solution uniformly where protonated chitosan was coated on the surface of MCFA in an acid solution. When the dispersion was adjusted to a basic environment, chitosan was precipitated to form MCFA@chitosan gel microcomposites (Support I). In the second strategy, MCFA/chitosan gel beads were prepared by the suspension method. With continuously mechanical stirring, the droplets containing MCFA and chitosan with a diameter in a range of a few hundred micrometers were stabilized by the emulsifier in the oil phase, forming unstable water-in-oil beads. After immersion in a mixture of NaOH solution and ethanol for 12 h, MCFA/chitosan gel beads that had a well-shaped spherical form with a porous structure (Support II) were obtained. The following immobilization process for Support I and Support II was identical by GDA activation method: First ionic exchange of cellulase on the GDA activated support, followed by the covalent attachment [42]. The amount of cellulase immobilized on Support I was 85.8 mg/g with an immobilization yield of 71.5%, whereas the amount of cellulase immobilized onto Support II was 76.8 mg/g with an immobilization yield of 64.0%. Both strategies are valid for cellulase immobilization, and the similar cellulase loading amount may be attributed to their different morphologies and particle sizes. Figure 2 shows the SEM images of the pristine MCFA, Support I and Support II prepared in this work. As shown in Figure 2a, the MCFA consisted of spherical particles with different size, along with smaller adhered particles or grains on the surface of the spherules. Support I presented non-porous spherical forms with little change in size compared with the pristine MCFA, but flocculent surface appeared because of chitosan coated on the surface of the MCFA (Figure 2b). The features presented by Support I are particularly attractive for cellulase immobilization due to its large surface area and plenty of amino groups presented in the chitosan, which could improve the immobilization capacity. Support II, consisting of lots of MCFA particles distributed separately in the chitosan gels, also showed spherical shape (Figure 2c). Although the size of Support II was 50 times larger than Support I, the amount of cellulase immobilized onto Support II was comparable with that of Support I. The porous structure as proven by the enlarged SEM image of Support II (Figure 2d) was responsible for the high loading amount. However, the activity recovery of these two examples of immobilized cellulase was quite different. The activity recoveries of Method I and Method II were 76.6% and 51.9%, respectively. The lower activity recovery of Method II was due to the difficulty in diffusion of substrate CMC to the internal of Method II.

### 3.2. Characterization of Immobilized Cellulase

**Figure 3:**
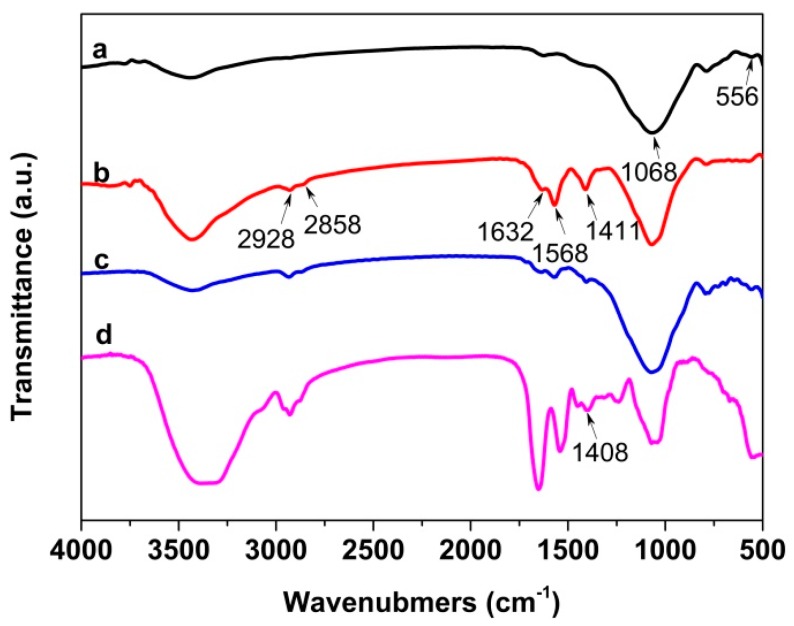
FT-IR spectra of (**a**) pristine MCFA, (**b**) Method I, (**c**) Method II and (**d**) free cellulase.

The chemical structure of pristine MCFA, immobilized cellulase and free cellulase were characterized and compared by FT-IR spectroscopy. As shown in Figure 3, the peak at 556 and 1068 cm^−1^ in the spectrum of MCFA was attributed to Fe–O bond and Al–O/Si–O asymmetric stretching vibrations, respectively [51,52]. The peak positions in the spectra of Method I and Method II were almost the same since they had identical chemical composition. Upon GDA activation, cellulase was covalent binding to the support by Schiff base linkage. Consequently, new absorption peaks at 2928 and 2858 cm^−1^ for the C–H stretching vibrations, peak at 1632 cm^−1^ for the C=N vibrations characteristic of imines, 1568 cm^−1^ for amide II of the peptide groups, and 1411 cm^−1^ for the characteristic band of proteins appeared in the spectra of Method I and Method II, indicating that cellulase immobilization was successful [53,54].

**Figure 4:**
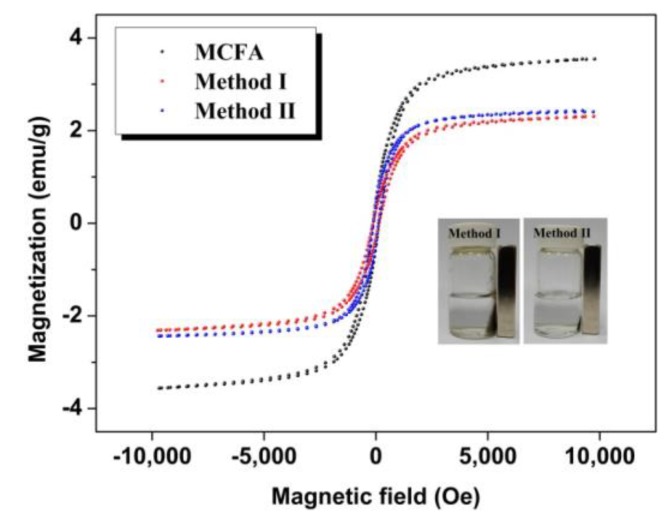
Magnetization curves of pristine MCFA, Method I and Method II.

The magnetization curves of pristine MCFA, Method I and Method II at room temperature are shown in Figure 4. The specific saturation magnetization of the pristine MCFA, Method I and Method II was 3.55, 2.31 and 2.43 emu/g, respectively. The combination of diamagnetic chitosan with the MCFA particles would decrease the specific saturation magnetization. Even so, both of these two immobilized cellulases could be conveniently recycled by magnetic separation, which is helpful for improving the reusability of immobilized cellulase. In particular, Method II is easier to recycle than Method I, which is attributed to its larger particle size.

### 3.3. Activity of Immobilized Cellulase

**Figure 5:**
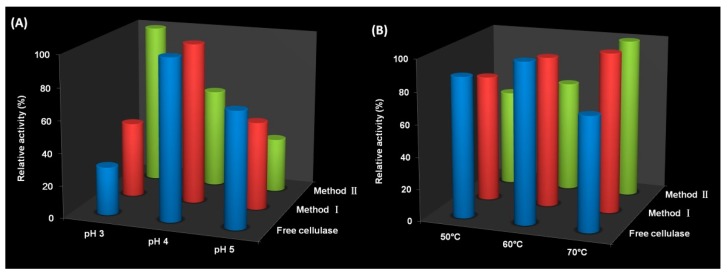
Effect of (**a**) pH and (**b**) temperature on activity of free cellulase, Method I and Method II.

The activity of free and immobilized cellulase at different pH and different temperatures were investigated because pH and temperature could influence the activity greatly. The activity in the pH range of 3−5 at 50 °C is plotted in Figure 5a. The result showed that optimum pH was 4 for both free cellulase and Method I, whereas this value shifted to 3 for Method II. Considering both immobilized cellulases have the same composition and immobilization protocol, the optimum pH should be the same. Their different morphologies may cause the difference of optimum pH for Method I and Method II. Support I presented non-porous spherical forms with a diameter of several micrometers and flocculent surface (Figure 2b). Support II showed a spherical shape with a diameter of several hundred micrometers and porous structure (Figure 2c,d). For Method II, the influence of pH on activity was more obviously because of its porous structure. As mentioned above, chitosan is protonated in an acid medium. The lower pH is conducive to expanding open channels due to the repulsion of ions which can promote the diffusion of substrate CMC to the internal of Method II. However, this result is unexpected for the above reason. In addition, the relative activity of both of these two immobilized cellulases was higher than that of free cellulase at pH 3 due to the presence of chitosan. Chitosan is protonated in an acid medium which has strong interactions with CMC. This means the concentration of CMC around the immobilized cellulase becomes higher under lower pH condition, which is conducive to promoting the hydrolysis reaction. Figure 5b shows the activity of free and immobilized cellulase in the range from 50−70 °C at pH 4. Free cellulase showed the highest activity at 60 °C and both of these two immobilized cellulases exhibited the highest activity at 70 °C. The higher optimum temperature of immobilized cellulase may be caused by multipoint covalent attachment which may enhance enzyme rigidity [55]. At 50 °C and 60 °C, Method II exhibited lower relative activity than Method I because diffusion of substrate CMC to Method II was much more difficult than to Method I at low temperature. Note that immobilized cellulase showed higher relative activity than free cellulase at 70 °C, indicating immobilized cellulase had preferable thermal adaptability.

Michaelis–Menten constant (K_m_) and maximum reaction velocity (V_max_) of free and immobilized cellulase were measured to know their kinetic parameters. The K_m_ of free cellulase, Method I and Method II was 7.607, 8.568 and 12.953 g/L, respectively. The V_max_ of free cellulase, Method I and Method II was 2.459 × 10^−2^, 2.185 × 10^−2^ and 1.961 × 10^−2^ g/(L min), respectively. The K_m_ and V_max_ of non-porous Method I were comparable with those of free cellulase. The highest K_m_ and lowest V_max_ of Method II may result from its porous structure, which will lead to the diffusion limitation of substrate.

**Figure 6:**
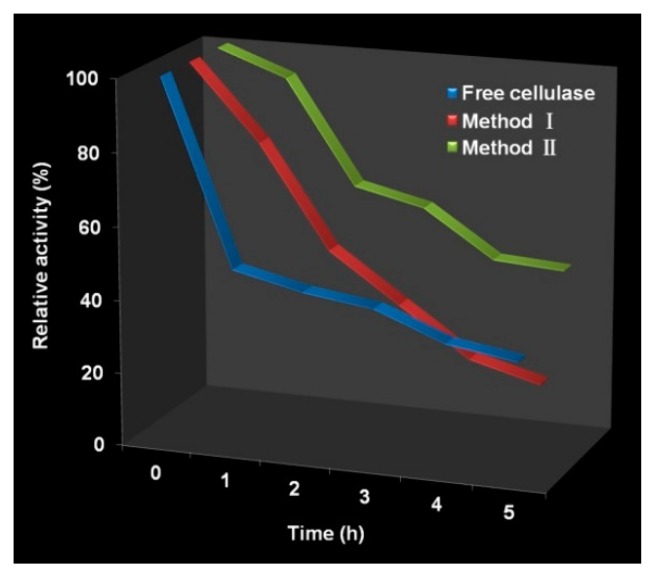
Thermal stability of free cellulase, Method I and Method II.

Figure 6 shows thermal stability of free and immobilized cellulase during 5 h at 70 °C. Both Method I and Method II had better relative activity than that of free cellulase during the first 2 h. However, the thermal stability of Method I decreased significantly, whereas Method II still kept excellent thermal stability. The enzyme half-life (t_1/2_) of free cellulase, Method I and Method II at 70 °C is 2.65, 2.13 and 3.96 h, respectively. The inactivation constant (k_d_) of free cellulase, Method I and Method II at 70 °C is 4.36 × 10^−3^, 5.42 × 10^−3^ and 2.92 × 10^−3^ h^−1^, respectively. Method II showed the best thermal stability and Method I exhibited the worst thermal stability. Their different morphologies may cause the difference of thermal stability. Multipoint covalent attachment of enzyme on GDA activated rigid solids can enhance enzyme rigidity to increase enzyme stability [8,42,43] and demonstrates one reason for the good thermal stability of Method II. Enzyme inactivation may occur by interactions with hydrophobic interfaces, and the porous structure of Method II can prevent this inactivation because only a very small amount of enzymes will be exposed to these hydrophobic interfaces [5,35,38,39]. As for Method I, though cellulase was immobilized on the solid support via multipoint covalent attachment as with Method II, the non-porous structure cannot prevent the inactivation mentioned above due to full exposure to the hydrophobic interfaces in stirred systems [5,38]. In addition, the small sizes of Method I make them easy to aggregate, leading to poor thermal stability. The improved thermal stability of Method II is of great importance to extending practical applications.

**Figure 7:**
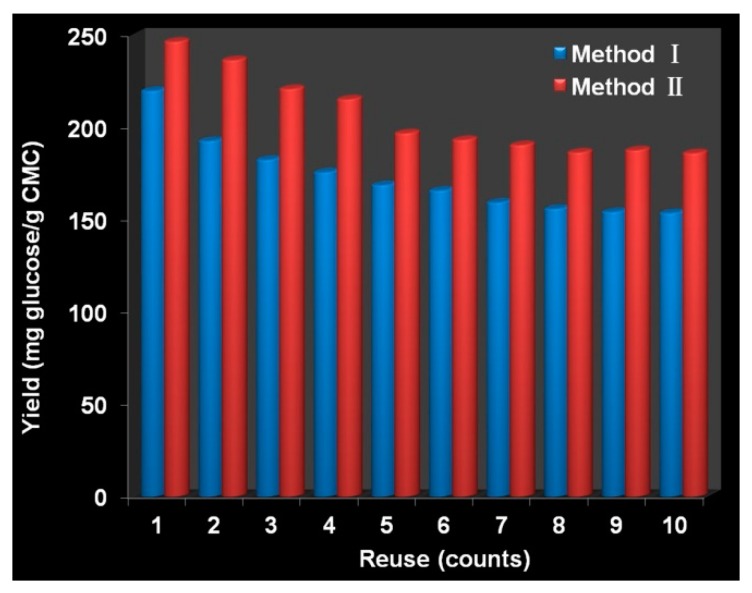
Reusability of immobilized cellulase.

The reusability of immobilized cellulase is also a key factor in the practical application. The two immobilized cellulases were used to hydrolyze 1% CMC and each cycle lasted 24 h. We can see from Figure 7 that the glucose productivity of immobilized cellulase during 24 h for each cycle showed the trend of slow decrease. Specifically for Method I, the glucose productivity was 219.8 mg glucose/g CMC and remained 69.9% of the original after 10 cycles; whereas the glucose productivity was 246.4 mg glucose/g CMC and kept 75.5% of its initial value after 10 repeated uses as for Method II. From the above data, we can know that Method II exhibited better reusability than Method I. Note that the size of porous Method II was much larger than that of Method I, but the glucose productivity was slightly better than that of Method I. Although diffusion of substrate CMC to Method II is much more difficult than to Method I, CMC could be hydrolyzed in the internal of Method II if given enough time. The decrease in glucose productivity may be caused by cellulase leakage during washing, end-product inhibition and enzyme inactivation [5,56,57].

## 4. Conclusions

Two kinds of support were successfully prepared by the combination of MCFA and chitosan where MCFA provided magnetism and chitosan played a significant part of increasing immobilization capability. The morphology and particle size of support has influences on activity recovery, optimum pH and temperature, thermal stability and reusability of immobilized cellulase. The non-porous MCFA@chitosan gel microcomposites with small particle size showed relative high immobilization yield and activity recovery, but poor thermal stability. The porous MCFA/chitosan gel beads with large particle size exhibited relative high thermal stability, but low immobilization yield and activity recovery. The reuse was easy to achieve by applying external magnetic fields to recover immobilized cellulase. Compared to cellulase immobilized on MCFA@chitosan gel microcomposites, the immobilized cellulase using MCFA/chitosan gel beads as support showed better glucose productivity and reusability. Based on these results, these two supports can be recommended as ideal supports for enzyme immobilization.
